# Emerging and re-emerging fatal viral diseases

**DOI:** 10.1038/s12276-021-00608-9

**Published:** 2021-05-06

**Authors:** Young Ki Choi

**Affiliations:** College of Medicine and Medical Research Institute, Chungbuk National University, Cheongju, Republic of Korea

**Keywords:** Viral infection, Infection

In the 21st century, newly identified pathogens, mostly zoonotic or vector-borne infectious agents, have consistently and relentlessly threatened public health and caused deadly outbreaks of global concern. “Emerging infectious disease” is a term used to describe previously unknown or known infectious diseases that have the potential to cause outbreaks^1^. By extension, emerging infections include those caused by pathogens that are already present in the environment but previously did not cause infection or evolve a selective advantage for infection in a new host species^2^. Many factors may contribute to the emergence of a new zoonotic disease. These include evolutionary progression of virological determinants, changes in human demographics and behavior, dramatic increases in movement by people and animals, and environmental factors, such as ecologic and climatologic factors^2,3^.

In this special issue, entitled “Emerging and Re-emerging Fatal Viral Diseases”, we focus on emerging RNA viruses, such as coronaviruses, as well as severe acute respiratory syndrome coronavirus 2 (SARS-CoV-2), influenza virus, and severe fever with thrombocytopenia syndrome virus (SFTSV); this issue will cover innate immune sensing and viral evasion strategies of coronaviruses, the evolution and future of influenza pandemic preparedness, and the latest findings regarding the emerging tick-borne SFTSV.

Humans are equipped with elaborate immune mechanisms to protect against harmful invading pathogens. The innate immune system constitutes the first line of defense against invading pathogens and is frequently targeted by immune-evading pathogens. Coronaviruses have evolved multiple strategies to evade host antiviral immunity. In hosts with immune naivety, distinct mechanisms associated with virulence, and pathogenicity include the perturbation of the host defense system by hindering antiviral innate immune responses through multiple viral inhibitory mechanisms. This results in successful viral transmission and adaptation to human hosts. Kasuga et al.^4^ summarizes the current knowledge about the mechanisms underlying host sensing and immune responses against coronavirus invasion, as well as the immune evasion strategies of coronaviruses.

Viral recognition by the host immune system results in the rapid production of type I and III interferons (IFNs), which are potent multifunctional cytokines that play an important role in host defense. Subsequently, IFNs and cytokines harmonize, resulting in a timely and balanced early immune response, which further activates host antiviral defense mechanisms by recruiting multiple immune cells to the site of viral infection. However, recent studies have reported that SARS-CoV-2 evades the host immune response by inducing a delayed interferon (IFN) response, which provides a window for uncontrolled viral replication. Inhibition and further dysregulation of type I and III IFN responses lead to hyperinflammation in coronavirus disease 2019 (COVID-19) patients. Moreover, it has been hypothesized that a delayed but exaggerated type I IFN response contributes to the severe progression of COVID-19. To further elucidate the immunosuppressive mechanism utilized by SARS-COV-2, Kim and Shin investigated the innate sensing mechanisms of this virus and the mechanism by which IFN inhibits SARS-CoV-2 replication^5^.

Influenza viruses are emerging viruses belonging to the Orthomyxoviridae family that infect many species^6^ and pose a perpetual global threat to humans. Due to continuous evolution, influenza has caused unpredictable and recurring pandemics for centuries. Since there is no way of predicting when and where a zoonotic pathogen will emerge, investigation at the first sign of emergence is particularly important. Thus, the World Health Organization established a global influenza surveillance network to monitor circulating influenza strains with pandemic potential in both humans and animals^7^. However, the occurrence of the 2009 H1N1 pandemic exposed a major weakness in the existing pandemic preparedness plan. Harrington et al.^8^ reviewed the history of influenza pandemic preparedness and proposed additional measures for the improvement of current influenza preparedness plans. In addition, they also address the intersection between the influenza pandemic preparedness network and the current SARS-CoV-2 crisis.

Contributing to the emergence of zoonotic viral diseases is the capacity of viruses to adapt to diverse econiches, such as adaptation to and transmission by arthropods. A tick-borne infectious disease, SFTS, is caused by a novel phlebovirus and is an increasing public health concern. SFTS was first identified in China in 2009, and epidemics have occurred in several East Asian countries since then. With the increasing incidence of SFTS and the rapid, worldwide spread of SFTSV vectors, this virus clearly has pandemic potential and presents an impending threat to global public health. Casel et al.^9^ provided the latest findings regarding SFTSV, including information on vector and virus transmission, genotype diversity and epidemiology, a probable pathogenic mechanism, and clinical manifestations of human SFTS. In addition, the development of experimental animal models to further understand SFTSV pathogenesis is also discussed.

The emergence of novel fatal viral diseases that pose continuous threats and impose constant global health challenges has fueled intense efforts to improve the understanding of virus molecular and cellular biology. However, despite concerted efforts to elucidate the complexities of these emerging viruses, scant new information regarding the ever-changing threats they pose exists. This special issue provides a comprehensive review of and insights into current trending topics regarding emerging viral diseases. It is of invaluable significance to understand the intricacies of host–virus interactions, which is necessary to overcome emerging fatal viral diseases and to prepare for potential pandemics that may occur in the near future.
